# Towards the Probabilistic Analysis of Small Bowel Capsule Endoscopy Features to Predict Severity of Duodenal Histology in Patients with Villous Atrophy

**DOI:** 10.1007/s10916-020-01657-9

**Published:** 2020-10-02

**Authors:** Stefania Chetcuti Zammit, Lawrence A Bull, David S Sanders, Jessica Galvin, Nikolaos Dervilis, Reena Sidhu, Keith Worden

**Affiliations:** 1 Academic Unit, Department of Gastroenterology, Sheffield Teaching Hospitals, Sheffield, UK; 2 Gastroenterology Department, Royal Hallamshire Hospital, Glossop Road, Sheffield, S102JF UK; 3 Dynamics Research Group, Department of Mechanical Engineering, University of Sheffield, Sheffield, UK; 4 Barts and The London School of Medicine and Dentistry, Queen Mary University of London, London, UK

**Keywords:** Celiac disease, Seronegative villous atrophy, Probabilistic analysis, Small bowel capsule endoscopy, Duodenal histology

## Abstract

Small bowel capsule endoscopy (SBCE) can be complementary to histological assessment of celiac disease (CD) and serology negative villous atrophy (SNVA). Determining the severity of disease on SBCE using statistical machine learning methods can be useful in the follow up of patients. SBCE can play an additional role in differentiating between CD and SNVA. De-identified SBCEs of patients with CD and SNVA were included. Probabilistic analysis of features on SBCE were used to predict severity of duodenal histology and to distinguish between CD and SNVA. Patients with higher Marsh scores were more likely to have a positive SBCE and a continuous distribution of macroscopic features of disease than those with lower Marsh scores. The same pattern was also true for patients with CD when compared to patients with SNVA. The validation accuracy when predicting the severity of Marsh scores and when distinguishing between CD and SNVA was 69.1% in both cases. When the proportions of each SBCE class group within the dataset were included in the classification model, to distinguish between the two pathologies, the validation accuracy increased to 75.3%. The findings of this work suggest that by using features of CD and SNVA on SBCE, predictions can be made of the type of pathology and the severity of disease.

## Introduction

Currently, the gold standard for the diagnosis of celiac disease (CD) and seronegative villous atrophy (SNVA) is based on duodenal histology [1]. Apart from the invasive nature of a gastroduodenoscopy, histological diagnosis can be flawed with errors. Unless an appropriate number of biopsies are taken and the samples are properly oriented [2] (at least four biopsies, including a biopsy from the duodenal bulb [1, 2]) a false negative result can occur. This is mainly because the mucosal distribution of CD is patchy [3–5]. Undiagnosed CD has long-term implications such as osteoporosis, iron deficiency anaemia, refractory celiac disease and SB malignancy [6].

CD and SNVA are histologically similar [7] and sometimes it is difficult to make a distinction between the two conditions based on other clinical and serological tests. Small bowel capsule endoscopy (SBCE) is carried out in patients with SNVA to assess for features of CD and to rule out other causes of villous atrophy [8]. Differentiating between CD and SNVA is important because of the different management these patients require. Patients with SNVA of unknown cause have been shown to respond to immunosuppressive therapy [9]. The mainstay of management of patients with CD is gluten-free diet (GFD) [10].

Determining severity of CD can be useful in the follow-up of patients as this enables comparison to be made [11]. Improving the diagnostic yield of SBCE through machine learning methods can help overcome some of these pitfalls in the diagnosis of CD and SNVA.

Machine learning for the detection of pathology on SBCEs has been previously explored, including to quantify aspects of macroscopic features of CD [12], for the detection of angieoectasias on SBCE [13], in the delineation of small bowel (SB) tumours [14], the recognition of inflammatory changes on SBCE [15] and even in the detection and assessment of colonic polyps on colon capsule endoscopy [16]. These studies have a common aim: to improve the delineation of pathology on SBCE. There is however, no literature on the use of machine learning methods to improve on the current reported sensitivities in the detection of pathology on SBCE based on human performance.

One aim was to assess whether a probabilistic model could be used to predict severity of duodenal histology in patients with CD and SNVA by considering features on SBCE. Another aim was to assess whether a similar model could be used to predict the type of disease (CD or SNVA) through macroscopic features on SBCE.

## Methodology

### Study design and patients

Patients with newly diagnosed and established CD and SNVA were included in this study over a one-year period from a tertiary centre for the management of CD. All patients had a confirmative diagnosis of CD or SNVA from serology and histology. Patients with SNVA had negative CD serology and were not on GFD at the time of histological diagnosis. They underwent extensive investigations to rule out other causes of SNVA such as inflammatory or infective conditions [9, 17]. All patients had a gastroduodenoscopy within two weeks from SBCE for duodenal histology and contemporary CD serology was checked. They underwent a SBCE to assess severity of disease, to rule out complications and to exclude other causes for SNVA such as Crohn’s disease.

SBCEs were de-identified and read by two expert reviewers (more than 300 capsules per year) who were blinded to each other’s findings, the indication for SBCE and the histology result from duodenal biopsies. To increase the size and variation, patients were considered as separate participants in the study if their SBCE was read differently by the two reviewers. After including the additional readings, the dataset included 81 sets of features (i.e. readings), corresponding to 72 original patients. This allowed for a larger dataset to be studied.

### Duodenal histology

At least two biopsies from the duodenal bulb and four biopsies from the second part of the duodenum were taken during gastroduodenoscopy. The histology was then classified according to the modified Marsh Criteria which reflects severity of changes [18] (Table 1). All histological samples were reviewed by two expert histopathologists. In the case of discrepancy, a third histopathologist was involved.

**Table 1:** Marsh classification of histological changes of celiac disease; *IEL: intraepithelial lymphocytes

Marsh Type	IEL* / 100 enterocytes – jejunum	IEL / 100 enterocytes - duodenum	Crypt hyperplasia	Villi
0	<40	<30	Normal	Normal
1	>40	>30	Normal	Normal
2	>40	>30	Increased	Normal
3a	>40	>30	Increased	Mild atrophy
3b	>40	>30	Increased	Marked atrophy
3c	>40	>30	Increased	Complete atrophy

### Small bowel capsule endoscopy

All patients underwent SBCE using Pillcam SB3 (Medtronic, Minneapolis, USA) [19].

**Fig. 1:**
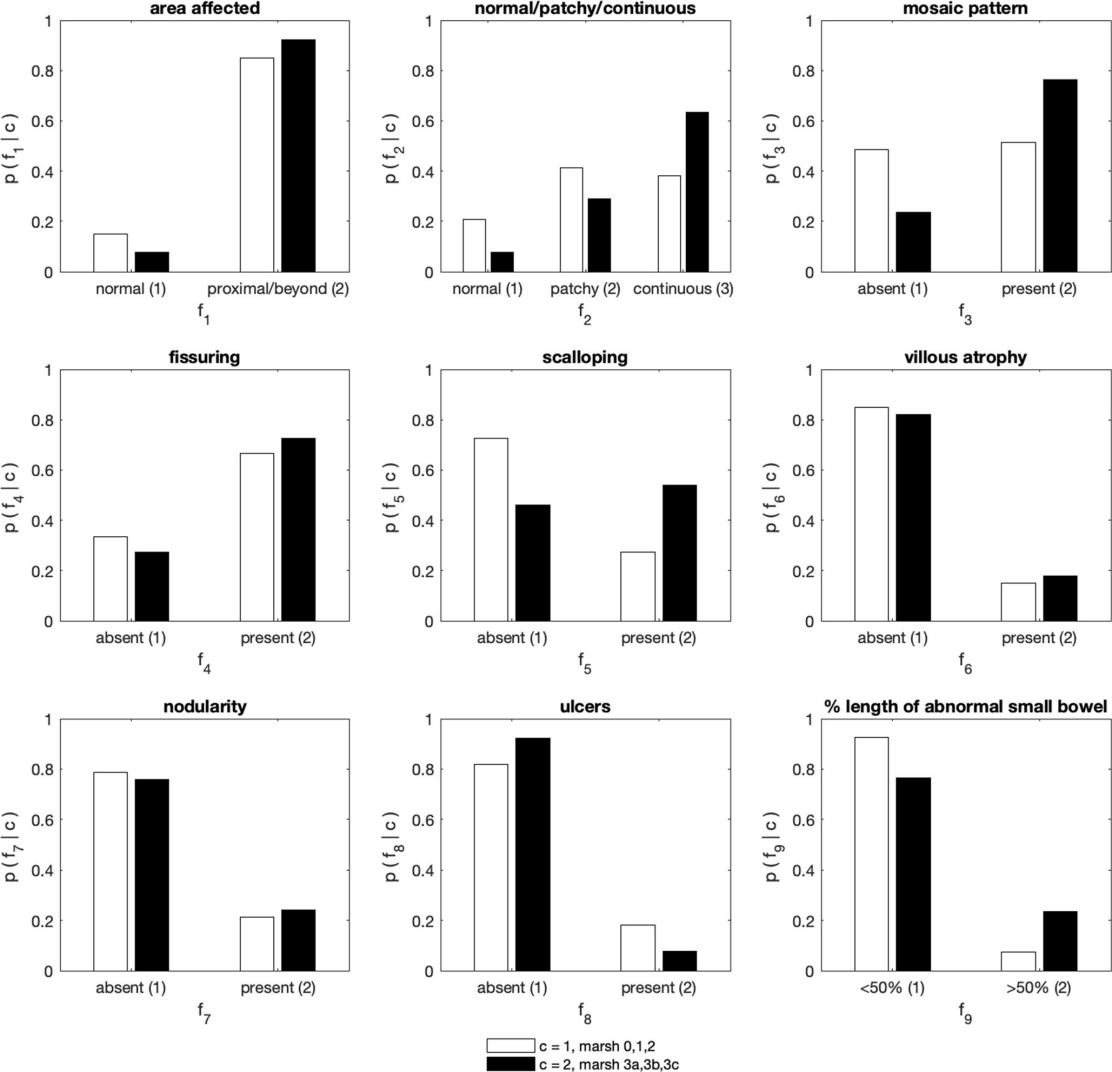
(a) fissuring of folds, (b) scalloping of folds, (c) villous atrophy, (d) mosaic pattern, (e) nodularity and (f) ulcers

Features reviewed included: total area affected, patchy /continuous pattern and macroscopic features of CD: mosaic pattern, fissuring of mucosa, scalloping of folds, villous atrophy, nodularity of mucosa and presence of ulcers (Fig. 1).

### Ethical considerations

The study protocol was approved by the Yorkshire and the Humber Research Ethics committee (IRAS 232382) and registered with the local research and development department of Sheffield Teaching Hospital NHS Foundation Trust under the registration number STH 19998. All SBCEs used in this study were de-identified. No additional consent was required for the study with the use of de-identified videos as assessed and approved formally by the Research Ethics Committee.

### Capsule endoscopy features

CD on SBCE was represented by nine features, {*f*_1_, …, *f*_9_}.

1 $$ SBCE=\left\{{f}_1,\dots, {f}_9\right\} $$

Each feature *f*_*i*_ was considered to be a categorical variable, with possible values *f*_*i*_ ∈ {1, …, *K*_*i*_}. These corresponded to the associated condition of that feature; thus, *f*_*i*_ ∈ {1, …, *K*_*i*_} (Table 2).

**Table 2:** SBCE feature descriptions

Feature, *f*_*i*_	Outcome, (*k*),
area affected, (*f*_1_)	normal (*k* = 1), proximal & beyond (*k* = 2)
normal/patchy/continuous, (*f*_2_)	normal (*k* = 1), patchy (*k* = 2), continuous (*k* = 3)
mosaic pattern, (*f*_3_)	absent (*k* = 1), present (*k* = 2)
fissuring, (*f*_4_)	absent (*k* = 1), present (*k* = 2)
scalloping, (*f*_5_)	absent (*k* = 1), present (*k* = 2)
villous atrophy, (*f*_6_)	absent (*k* = 1), present (*k* = 2),
nodularity, (*f*_7_)	absent (*k* = 1), present (*k* = 2)
ulcers, (*f*_8_)	absent (*k* = 1), present (*k* = 2)
% length of abnormal small bowel mucosa, (*f*_9_)	<50% (k = 1), > 50% (k = 2)

### Target predictions

The data were used to build two predictive models. The class associated with each patient was defined by the value of the variable *c*. The first model predicted mild (*c* = 1) or severe Marsh scores (*c* = 2). The aim of the second model was to differentiate between patients with SNVA (*c* = 1), and CD (*c* = 2). Two, two-class classifiers were defined, where *c* ∈ {1, 2} (Tables 3 and 4).

**Table 3:** Mild vs Severe Marsh Scores

Class, *c*	Description	Number of Readings
*c* = 1	Mild Marsh scores (Marsh score 0, 1, 2)	31
*c* = 2	Severe Marsh scores (Marsh score 3a, 3b, 3c)	50

**Table 4:** CD (celiac disease) vs SNVA (seronegative villous atrophy)

Class, *c*	Description	Number of Readings
*c* = 1	SNVA	18
*c* = 2	CD	63

### Probabilistic analysis of features

Following a probabilistic approach, each feature *f*_*i*_ was considered to be categorically distributed,

2 $$ p\left({f}_i=k|c\right)= Cat\left({f}_i\right|{\lambda}_i\Big) $$

The parameter *λ*_*i*_ can be considered to be a histogram over the *K* possible conditions for the feature *f*_*i*_ (in the class *c*); therefore, *k* ∈ {1, …, *K*} and *λ*_*i*_ = {*λ*_*i*1_, …, *λ*_*iK*_}. Specifically, the probability of condition *k* was,

3 $$ p\left({f}_i=k\right)={\lambda_i}_k $$

In words, *p*(*f*_*i*_ = *k*| *c*) was the likelihood that the condition of feature *f*_*i*_ was equal to *k*, given the class *c*.Each feature was considered to be conditionally independent. This assumption is appropriate for smaller datasets, as there are less parameters to learn from the data. The assumption of conditional independence (between features) is common for Naïve Bayes classification, which is shown to be appropriate in many applications, even if correlation between the features is expected [20]. The likelihood of a SBCE given the class *c* was the product of the likelihoods of its corresponding features,

4 $$ p\left( SBCE|c\right)=\prod \limits_{i=1}^9p\left({f}_i=k|c\right) $$

such that *p*(*SBCE*| *c*) was the likelihood of observing a SBCE, given the patient was in class *c*. For example, the likelihood of a given SBCE feature-set, given that the patient was in the severe Marsh score group.

To inform feature analysis, and to make predictions, the parameters of the distribution (for each feature) were learnt from the available data. In this work, a Bayesian estimate of the parameters was used, to mitigate overtraining, and account for the zero-count problem [20, 21]. Additionally, a Bayesian approach leads to distributions over the predicted values, allowing for the uncertainty associated with predictions to be approximated. To calculate Bayesian estimates, a prior distribution was placed over the parameters of the categorical distribution, *λ*_*i*_, for each feature. The parameters were then marginalised out (from the model) by integration. An appropriate prior was the Dirichlet distribution, as it was compatible to and conjugate with the categorical distribution [21]. Conjugacy is desirable, as it leads to tractable solutions (the functional form of the posterior distribution will be the same as the prior). The distribution is specified by,

5 $$ p\left({\lambda}_i\right)= Dir\left({\lambda}_i|\alpha \right) $$

6 $$ \alpha =\left\{{\alpha}_1,\dots, {\alpha}_K\right\} $$

The parameters of the Dirichlet distribution, α, were set to 1 (for conditions 1 to κ) for each feature. In terms of regularisation, this corresponded to add-one or Laplace smoothing [20]. As discussed, the effects of the parameters were then integrated out, to provide the posterior-predictive distribution. In this case, this was a posterior-predictive likelihood,

7 $$ p\left({f}_i=k|c\right)=\int p\left({f}_i|c,{\lambda}_i\right)p\left({\lambda}_i\right) d\lambda $$

8 $$ p\left({f}_i=k|c\right)=\frac{\left({N}_k+{\alpha}_k\right)}{N+{\sum}_{k=1}^K{\alpha}_k} $$

*N* was considered to be the total number of patients and *N*_*k*_ was the number of patients (in class *c*), with the value *k* for the feature *f*_*i*_. The likelihood *p*(*f*_*i*_| *c*) was the (estimated) proportion of the patients in class *c* with condition *k* for feature *f*_*i*_; for example, the proportion of patients in the dataset with a severe Marsh score (*c* = 2), who showed (*k* = 2) scalloping (*f*_5_).

## Results

### Characteristics of the cohort studied

Seventy-two patients (45; 62.5% females, mean age 52.5 ± 16.6 years) were included in this study. Patients had a diagnosis of CD (51, 70.8%) or SNVA (21, 29.2%). Marsh histology is shown in Table 5. A small proportion of patients (*n* = 14; 19%) had extensive abnormal small bowel mucosa beyond the proximal area. Seventeen patients (24%) had a normal SBCE. Patients had the following features of CD on SBCE: 30 (42%) scalloping, 42 (58%) fissuring, 38 (53%) mosaicism, 14 (19%) villous atrophy, 12 (17%) nodularity, 2 (3%) ulcers (Table 6).

**Table 5:** Marsh classification of disease

Marsh score	N (%)
0	9 (12.5)
1	15 (20.8)
2	3 (4.2)
3a	8 (11.1)
3b	19 (26.4)
3c	18 (25.0)

**Table 6:** Features of celiac disease on SBCE

Feature of CD* on SBCE**	Scalloping n (%)	Fissuring n (%)	Mosaicism n (%)	Villous atrophy n (%)	Nodularity n (%)	Ulcers n (%)	Absence of features n (%)***
	30 (42)	42 (58)	38 (53)	14 (19)	12 (17)	2 (3)	17 (24)
Extent of abnormal small bowel mucosa	Proximal n (%)		Beyond proximal n (%)		Normal n (%)		
	41 (57)		14 (19)		17 (24)		
Patchy / continuous pattern /normal	Patchy n (%)		Continuous n (%)		Normal n (%)		
	8 (11)		47 (65)		17 (24)		

*Celiac disease

**Small bowel capsule endoscopy

***Features do not add up to 100% as each patient could have more than one feature of celiac disease on small bowel capsule endoscopy

### Feature analysis–severity of marsh classification of disease

**Fig. 2:**
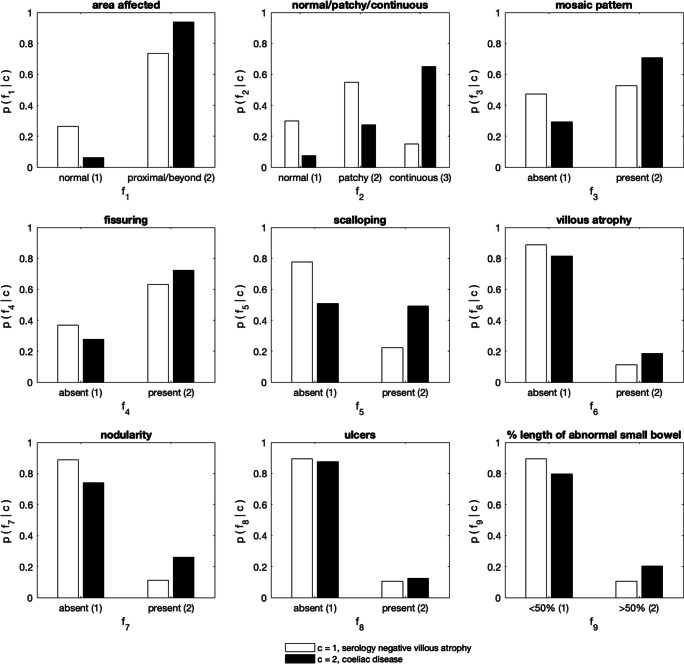
Histograms learnt from the data, showing the likelihood of each feature, given the class of patients corresponding to: mild (white), or severe (black) Marsh score

The posterior-predictive likelihoods for the features associated with mild vs severe Marsh scores are illustrated in Fig. 2. The likelihoods can be interpreted as the histogram over the possible conditions for each feature, given the class of patients, and the information in all the available data. The likelihoods showed that all features (other than *f*_8_ for the Marsh score model) were likely to represent more severe disease, i.e. *k* > 1, for:

- severe Marsh scores (rather than mild)
- CD (rather than SNVA).

Patients with higher Marsh scores were more likely to have a positive SBCE and a continuous distribution of macroscopic features (Fig. 2). Features including mosaic pattern, fissuring and scalloping of folds were indicative of a more severe Marsh classification. Villous atrophy and nodularity of mucosa were more common in patients with severe Marsh scores but the difference was not distinct. Ulcers were present in a small number of patients, and frequencies were similar in both groups. Overall, most patients had less than 50% of the SB involved, and those with more severe Marsh scores had more extensive SB involvement.

### Feature analysis – Type of disease (SNVA or CD)

**Fig. 3:**
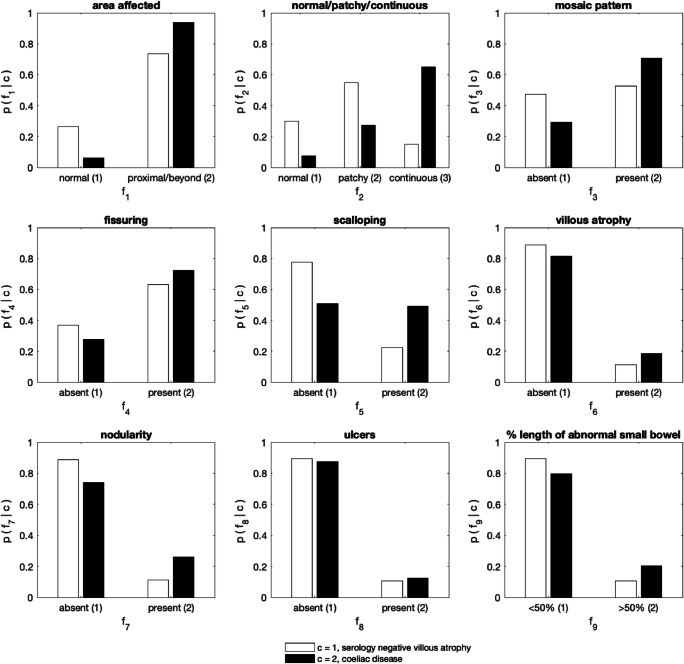
Histograms learnt from the data, showing the likelihood of each feature, given the class corresponding to: serology negative villous atrophy (white), or celiac disease (black)

The posterior-predictive likelihoods for SNVA and CD are shown in Fig. 3. Again, the likelihoods can be interpreted as the histogram over the possible conditions for each feature, given the class of patients, and the information in all the available data. Fig. 3 illustrates that patients with CD were more likely to have a positive SBCE than those with SNVA. Patients with CD were more likely to have a continuous distribution of features. Patients with SNVA were more likely to have patchy disease. Features such as mosaic pattern of the mucosa, fissuring of folds, scalloping, villous atrophy and nodularity of mucosa were more likely to be present in patients with CD than patients with SNVA. Patients with CD were more likely to have extensive SB involvement.

### Predictive model: Maximum-likelihood

A maximum likelihood approach was used to define a model for prediction. Specifically, the predicted class, $\hat{c_{\ast }}$ is the class with the maximum posterior-predictive likelihood for the *SBCE*_∗_,

9 $$ \hat{c_{\ast }}= argma{x}_{c\in \left\{1,2\right\}}\left\{\ p\left( SBC{E}_{\ast}\right|c\right)\Big\} $$

The performance of each classifier was assessed using Leave-One-Out cross-validation (LOOCV) [22]. This involved learning the parameters of the likelihood model (Eq. 8) with all of the available data and excluding one patient (the validation data). The model was then used to predict the class of the single, held-out patient, and a score was recorded. The score was considered unity if the patient was successfully classified and zero if the patient was misclassified. This process was repeated, such that all 81 SBCE readings were held out for validation in turn. Finally, the score over the whole data set was presented as a (percentage) accuracy.

Following LOOCV, the validation accuracy was 69.1% when predicting the severity of the Marsh score (Table 3) and when distinguishing between CD and SNVA (Table 4).

### Predictive model: Naïve Bayes

To estimate the probability of each class given the SBCE of a patient, the distribution over *c* could be estimated and included in the model. To include this information, *p*(*c*) was estimated from the data. In other words, the probability of observing a class, *c*, was estimated, given the number of times it occurred in the dataset (in a similar manner to the feature analysis). Therefore,

10 $$ p(c)= Cat\left(c|\pi \right) $$

The parameter *π* was considered to be a histogram over the two possible classes (for each predictor), *c* ∈ {1, 2}.Thus, *π* = {*π*_1_, *π*_2_}, and,

11 $$ p\left(c=i\right)={\pi}_i $$

Again, a Dirichlet prior was placed over *π*, and the *α* parameter was set to a vector of ones, corresponding to add-one (Laplace) smoothing. The marginal probability over classes could then be estimated by,

12 $$ p(c)=\frac{\left({N}_c+1\right)}{N+2} $$

Note, *N* was the total number of SBCE readings, and *N*_*c*_ was the number of patients in class *c*. A probabilistic classifier could then be defined using Bayes rule [20], which was used to define a posterior-predictive distribution over the class groups, given the features from the SBCE of a patient,

13 $$ p\left({c}_{\ast }| SBC{E}_{\ast}\right)=\frac{p\left( SBC{E}_{\ast }|\ {c}_{\ast}\right)\ p\left({c}_{\ast}\right)}{\sum_{c_{\ast }=1}^2p\left( SBC{E}_{\ast }|\ {c}_{\ast}\right)\ p\left({c}_{\ast}\right)} $$

Therefore, *p*(*c*_∗_| *SBCE*_∗_) was a two-dimensional histogram, corresponding to the probability of a patient belonging to:

- mild Marsh scores or severe Marsh scores;
- SNVA or CD.

Importantly, the success of this approach requires that the proportion of patients in each class is representative of future data.

Firstly, the estimate of *p*(*c*) was included in the model used to predict Marsh scores. In this case, the LOOCV accuracy decreased significantly to 56.8%. The probability *p*(*c*) was also included into the model to predict differentiation between classes of SNVA and CD. For this predictor, the validation accuracy increased to 75.3%.

## Discussion

This is the first study that demonstrates how macroscopic features of CD/SNVA on SBCE can help predict the severity of disease. It is also the first study to show that pattern recognition on SBCE can help distinguish between CD and SNVA.

In patients with newly-diagnosed CD, severity of duodenal histology at the time of diagnosis can be predictive of histological recovery at one year from diagnosis [23]. In established CD, incomplete mucosal recovery can also be predictive of complications [24]. Predicting severity of disease by considering features on SBCE can help physicians assess the risk of complications or predict the time needed for mucosal recovery. Quantifying severity of CD on SBCE is useful in the follow up of patients with CD, as this enables a comparison to be made between SBCEs before and after treatment is commenced [11, 25]. Patients with persistently similar features on follow up SBCE can be managed more aggressively by adding immunosuppressants. In this study, patients with higher Marsh scores were more likely to have macroscopic evidence of CD and for a longer distribution on SBCE.

There are several causes of SNVA such as medications, infections and inflammatory conditions [17]. These can cause histological changes that can mimic CD. CD and SNVA cannot be distinguished by considering histology only, but by taking into consideration other parameters such as human leucocyte antigen genotype, CD serology and exclusion of other causes of villous atrophy. Recognition of differences in patterns of disease in the SB for both conditions can enable SBCE to play an additional role in their distinction. Differentiating between both conditions is crucial because of different management [26] and also because of the higher mortality associated with SNVA than with CD [27]. In the second model, that predicted the distinction between CD and SNVA, patients with CD were more likely to have macroscopic evidence of disease on SBCE than patients with SNVA.

The sensitivity of SBCE to detect features of CD varies between 71 and 93% [25, 28–33]. For both models, a LOOCV validation accuracy of 69.1% was achieved using a maximum (posterior-predictive) likelihood approach. While these accuracies may seem low, the performance is near the quoted sensitivity of the SBCE, which effectively limits the classification accuracy of the model.

The reliability of the probabilistic model relies on the assumption that the number of patients in each class is representative of future data. When the proportion of patients was taken into consideration in the severity Marsh score model, the LOOCV accuracy decreased to 56.8%; therefore, it is assumed that the available data were not representative of the expected distribution over the class labels *p*(*c*) for marsh score subgroups (mild / severe). For CD or SNVA, however, the validation accuracy increased to 75.3% by including an estimate of the distribution over class marginal, used to define Naïve Bayes classifier. The performance increase for the CD SNVA model makes sense, as the estimate of the distribution *p*(*c*) (77.8% CD, 22.2% SNVA) was similar to that reported in the literature (77% CD, 29% SNVA) in a group of patients with varying villous atrophy [34].

One limitation of the study was the relatively small dataset, however, this is one of the largest CD/SNVA SBCE dataset compared to the current literature. Another limitation of this study is the lack of distinction between SNVA-CD, SNVA in patients with an unknown cause and SNVA in those with an identifiable cause other than CD; however, further classification into these subgroups would have rendered the groups even smaller.

## Conclusions

Using probabilistic analysis of macroscopic features on SBCE it is suggested that more pronounced and extensive features are present in patients with more severe Marsh scores of histology and in patients with CD (as opposed to SNVA). The findings of this work suggest that, from the available data, SBCE features are suitable in making predictions as to whether a patient has CD or SNVA and to determine the severity of disease. With validation on new patients, this implies that the diagnosis of CD or SNVA and the severity of disease may be supported by probabilistic machine learning, applied to SBCE features.

## References

[1] Ludvigsson JF, Bai JC, Biagi F, Card TR, Ciacci C, Ciclitira PJ, Green PH, Hadjivassiliou M, Holdoway A, van Heel DA, et al.: Diagnosis and management of adult coeliac disease: guidelines from the British Society of Gastroenterology. *Gut*, 63:1210–1228, 2014. 10.1136/gutjnl-2013-306578 PMC4112432 24917550

[2] Collin P, Kaukinen K, Vogelsang H, Korponay-Szabo I, Sommer R, Schreier E, Volta U, Granito A, Veronesi L, Mascart F, et al.: Antiendomysial and antihuman recombinant tissue transglutaminase antibodies in the diagnosis of coeliac disease: a biopsy-proven European multicentre study. *Eur J Gastroenterol Hepatol*, 17:85–91, 2005. 10.1097/00042737-200501000-00017 15647647

[3] Pais WP, Duerksen DR, Pettigrew NM, Bernstein CN: How many duodenal biopsy specimens are required to make a diagnosis of celiac disease? *Gastrointestinal endoscopy*, 67:1082–1087, 2008. 10.1016/j.gie.2007.10.015 18308317

[4] Green PH: Celiac disease: how many biopsies for diagnosis? *Gastrointestinal endoscopy*, 67:1088–1090, 2008. 10.1016/j.gie.2007.12.035 18513550

[5] Hopper AD, Cross SS, Sanders DS: Patchy villous atrophy in adult patients with suspected gluten-sensitive enteropathy: is a multiple duodenal biopsy strategy appropriate? *Endoscopy*, 40:219–224, 2008. 10.1055/s-2007-995361 18058655

[6] Majsiak E, Cichoz-Lach H, Gubska O, Cukrowska B: [Celiac disease - disease of children and adults: symptoms, disease complications, risk groups and comorbidities]. *Pol Merkur Lekarski*, 44:31–35, 2018. 29374421

[7] Arévalo Suárez F, Portugal S, Barreda C, Montes P, Perez-Narrea MT, Rodríguez O, Vergara G, Monge E: [Celiac disease and negative serology villous atrophy: histological comparison and immunohistochemical study of CD3, CD4, CD8 and CD56 lymphocytes]. *Rev Gastroenterol Peru*, 36:123–128, 2016. 27409088

[8] Pennazio M, Spada C, Eliakim R, Keuchel M, May A, Mulder CJ, Rondonotti E, Adler SN, Albert J, Baltes P, et al.: Small-bowel capsule endoscopy and device-assisted enteroscopy for diagnosis and treatment of small-bowel disorders: European Society of Gastrointestinal Endoscopy (ESGE) Clinical Guideline. *Endoscopy*, 47:352–376, 2015. 10.1055/s-0034-1391855 25826168

[9] DeGaetani M, Tennyson CA, Lebwohl B, Lewis SK, Abu Daya H, Arguelles-Grande C, Bhagat G, Green PH: Villous atrophy and negative celiac serology: a diagnostic and therapeutic dilemma. *Am J Gastroenterol*, 108:647–653, 2013. 10.1038/ajg.2013.45 23644957

[10] Al-Toma A, Volta U, Auricchio R, Castillejo G, Sanders DS, Cellier C, Mulder CJ, Lundin KEA: European Society for the Study of Coeliac Disease (ESsCD) guideline for coeliac disease and other gluten-related disorders. *United European Gastroenterol J*, 7:583–613, 2019. 10.1177/2050640619844125 PMC6545713 31210940

[11] Chetcuti Zammit S, Sanders DS, Cross SS, Sidhu R: Capsule endoscopy in the management of refractory coeliac disease. *J Gastrointestin Liver Dis*, 28:15–22, 2019. 10.15403/jgld.2014.1121.281.cel 30851167

[12] Zhou T, Han G, Li BN, Lin Z, Ciaccio EJ, Green PH, Qin J: Quantitative analysis of patients with celiac disease by video capsule endoscopy: A deep learning method. *Comput Biol Med*, 85:1–6 2017. 10.1016/j.compbiomed.2017.03.031 28412572

[13] Tsuboi A, Oka S, Aoyama K, Saito H, Aoki T, Yamada A, Matsuda T, Fujishiro M, Ishihara S, Nakahori M, et al.: Artificial intelligence using a convolutional neural network for automatic detection of small-bowel angioectasia in capsule endoscopy images. *Dig Endosc* 2019. 10.1111/den.13507 31392767

[14] Vieira PM, Freitas NR, Valente J, Vaz IF, Rolanda C, Lima CS: Automatic Detection of Small Bowel Tumors in Wireless Capsule Endoscopy Images Using Ensemble Learning. *Med Phys* 2019. 10.1002/mp.13709 31299096

[15] Aoki T, Yamada A, Aoyama K, Saito H, Tsuboi A, Nakada A, Niikura R, Fujishiro M, Oka S, Ishihara S, et al.: Automatic detection of erosions and ulcerations in wireless capsule endoscopy images based on a deep convolutional neural network. *Gastrointest Endosc*, 89:357–363.e352, 2019. 10.1016/j.gie.2018.10.027 30670179

[16] Blanes-Vidal V, Baatrup G, Nadimi ES: Addressing priority challenges in the detection and assessment of colorectal polyps from capsule endoscopy and colonoscopy in colorectal cancer screening using machine learning. *Acta Oncol*, 58:S29–S36, 2019. 10.1080/0284186X.2019.1584404 30836800

[17] Aziz I, Peerally MF, Barnes JH, Kandasamy V, Whiteley JC, Partridge D, Vergani P, Cross SS, Green PH, Sanders DS: The clinical and phenotypical assessment of seronegative villous atrophy; a prospective UK centre experience evaluating 200 adult cases over a 15-year period (2000-2015). *Gut*, 66:1563–1572, 2017. 10.1136/gutjnl-2016-312271 27605538

[18] Oberhuber G, Granditsch G, Vogelsang H: The histopathology of coeliac disease: time for a standardized report scheme for pathologists. *Eur J Gastroenterol Hepatol*, 11:1185–1194, 1999. 10.1097/00042737-199910000-00019 10524652

[19] Zwinger LL, Siegmund B, Stroux A, Adler A, Veltzke-Schlieker W, Wentrup R, Jürgensen C, Wiedenmann B, Wiedbrauck F, Hollerbach S, et al.: CapsoCam SV-1 Versus PillCam SB 3 in the Detection of Obscure Gastrointestinal Bleeding: Results of a Prospective Randomized Comparative Multicenter Study. *J Clin Gastroenterol* 2018. 10.1097/MCG.0000000000000994 29369240

[20] P. MK: *Machine Learning: a Probabilistic Perspective.* . MIT press; 2012.

[21] Gelman A. SHS, Carlin J. B., Dunson D. B., V ehtari A., Rubin D. B . *Bayesian Data Analysis* Chapman and Hall/CRC; 2013.

[22] BCM: *Pattern Recognition and Machine Learning.* . Springer; 2006.

[23] Pekki H, Kurppa K, Mäki M, Huhtala H, Sievänen H, Laurila K, Collin P, Kaukinen K: Predictors and Significance of Incomplete Mucosal Recovery in Celiac Disease After 1 Year on a Gluten-Free Diet. *Am J Gastroenterol*, 110:1078–1085, 2015. 10.1038/ajg.2015.155 26032154

[24] Larussa T, Suraci E, Imeneo M, Marasco R, Luzza F: Normal Bone Mineral Density Associates with Duodenal Mucosa Healing in Adult Patients with Celiac Disease on a Gluten-Free Diet. *Nutrients*, 9, 2017. 10.3390/nu9020098 PMC5331529 28146115

[25] Murray JA, Rubio-Tapia A, Van Dyke CT, Brogan DL, Knipschield MA, Lahr B, Rumalla A, Zinsmeister AR, Gostout CJ: Mucosal atrophy in celiac disease: extent of involvement, correlation with clinical presentation, and response to treatment. *Clin Gastroenterol Hepatol*, 6:186–193; 2008, quiz 125. 10.1016/j.cgh.2007.10.012 PMC2577378 18096440

[26] Schiepatti A, Sanders DS, Zuffada M, Luinetti O, Iraqi A, Biagi F: Overview in the clinical management of patients with seronegative villous atrophy. *Eur J Gastroenterol Hepatol*, 31:409–417, 2019. 10.1097/MEG.0000000000001340 30557227

[27] Schiepatti A, Biagi F, Fraternale G, Vattiato C, Balduzzi D, Agazzi S, Alpini C, Klersy C, Corazza GR: Short article: Mortality and differential diagnoses of villous atrophy without coeliac antibodies. *Eur J Gastroenterol Hepatol*, 29:572–576, 2017. 10.1097/MEG.0000000000000836 28350748

[28] El-Matary W, Huynh H, Vandermeer B: Diagnostic characteristics of given video capsule endoscopy in diagnosis of celiac disease: a meta-analysis. *Journal of laparoendoscopic & advanced surgical techniques.Part A*, 19:815–820, 2009. 10.1089/lap.2008.0380 19405806

[29] Hopper AD, Sidhu R, Hurlstone DP, McAlindon ME, Sanders DS: Capsule endoscopy: an alternative to duodenal biopsy for the recognition of villous atrophy in coeliac disease? *Digestive and liver disease : official journal of the Italian Society of Gastroenterology and the Italian Association for the Study of the Liver*, 39:140–145, 2007. 10.1016/j.dld.2006.07.017 16965945

[30] Lujan-Sanchis M, Perez-Cuadrado-Robles E, Garcia-Lledo J, Juanmartinena Fernandez JF, Elli L, Jimenez-Garcia VA, Egea-Valenzuela J, Valle-Munoz J, Carretero-Ribon C, Fernandez-Urien-Sainz I, et al.: Role of capsule endoscopy in suspected celiac disease: A European multi-centre study. *World journal of gastroenterology*, 23:703–711, 2017. 10.3748/wjg.v23.i4.703 PMC5292345 28216978

[31] Petroniene R, Dubcenco E, Baker JP, Ottaway CA, Tang SJ, Zanati SA, Streutker CJ, Gardiner GW, Warren RE, Jeejeebhoy KN: Given capsule endoscopy in celiac disease: evaluation of diagnostic accuracy and interobserver agreement. *Am J Gastroenterol*, 100:685–694, 2005. 10.1111/j.1572-0241.2005.41069.x 15743369

[32] Rokkas T, Niv Y: The role of video capsule endoscopy in the diagnosis of celiac disease: a meta-analysis. *European journal of gastroenterology & hepatology*, 24:303–308, 2012. 10.1097/MEG.0b013e32834fa914 22266837

[33] Rondonotti E, Spada C, Cave D, Pennazio M, Riccioni ME, De Vitis I, Schneider D, Sprujevnik T, Villa F, Langelier J, et al.: Video capsule enteroscopy in the diagnosis of celiac disease: a multicenter study. *Am J Gastroenterol*, 102:1624–1631, 2007. 10.1111/j.1572-0241.2007.01238.x 17459022

[34] Abrams JA, Diamond B, Rotterdam H, Green PH: Seronegative celiac disease: increased prevalence with lesser degrees of villous atrophy. *Dig Dis Sci*, 49:546–550, 2004. 10.1023/b:ddas.0000026296.02308.00 15185855

